# Vascular Endothelial Growth Factor (VEGF) and VEGF Receptor Inhibitors in Health and Disease

**DOI:** 10.3390/ph17070959

**Published:** 2024-07-18

**Authors:** Sylvain Broussy

**Affiliations:** CiTCoM, UMR CNRS 8038, U1268 INSERM, UFR de Pharmacie, Faculté de Santé, Université Paris Cité, 75006 Paris, France; sylvain.broussy@u-paris.fr

In this Special Issue of *Pharmaceuticals,* we present four reviews and seven original articles addressing recent aspects of research on Vascular Endothelial Growth Factors (VEGFs) and their receptors, from clinical practice to fundamental studies in new drug development. The VEGF family of cytokines constitutes the main pro-angiogenic and lymphangiogenic factors. In mammals, this family includes VEGF-A (or simply VEGF), VEGF-B, -C, -D, and placental growth factor (PlGF). Their intracellular effects are mediated by binding with three receptors, VEGFR-1/2/3, and two co-receptors, the neuropilins NRP-1/2. Over the past 20 years, pharmaceuticals targeting the VEGF/VEGFR axis (primarily VEGF-A/VEGFR-2) and its downstream signaling pathways have been successfully used to treat pathological angiogenesis, which plays a role in several cancers and ocular diseases. However, these treatments have their limitations, particularly with regard to their long-term efficiency and their route of administration [1,2]. 

In the VEGF/VEGFR axis, it is important to distinguish two biological targets and, therefore, two completely different types of pharmaceutical inhibitors. The first biological targets are extracellular: VEGF/VEGFR and VEGF/NRP interactions, which are protein/protein interaction interfaces, typically inhibited by large molecules. The second targets are the intracellular tyrosine kinase domains of VEGFRs and other downstream intracellular enzymes. These enzyme active sites are generally inhibited by small molecules. 

In clinical practice, extracellular VEGF ligands are classed as macromolecules, such as receptor fragments and antibodies. The anti-VEGF antibody bevacizumab was approved to treat colorectal cancer in 2004 and has since been widely used to treat other types of cancer. It is prescribed to treat non-small cell lung cancer in combination with paclitaxel–carboplatin chemotherapy [3]. The development of angiogenesis inhibitors in this pathology is the subject of intense research [4]. It was found that recombinant endostatin interferes with the VEGF/VEGFR axis and creates a “vascular normalization” window. In light of this, combining endostatin and radiotherapy appears to be a promising approach in non-small cell lung cancer (reviewed in Contribution 1). For ocular diseases, recent clinical studies continue to accurately evaluate the effects of intravitreal bevacizumab and ranibizumab, particularly for neovascular Age-Related Macular Degeneration (nAMD) (reviewed in Contribution 2). These studies confirm that these treatments have beneficial effects for the vast majority of patients (Contribution 3) and patient-reported outcomes indicated a significant improvement in quality of life (Contribution 4). However, administration of these macromolecules requires repeated intravitreal injections, leading to patient discomfort and, potentially, non-compliance with treatment. Interestingly, recent research on inhibitors of extracellular VEGF/VEGFR and VEGF/NRP interactions has expanded to smaller molecular entities with specifically designed properties, such as monospecific [5,6] and bispecific nanobodies [7,8], peptides, and peptidomimetics (reviewed in Contribution 5). These new potential therapeutic molecules include peptide ligands of VEGF [9,10] or VEGFRs (Contribution 6 and reference [11]) and peptidomimetics targeting NRP-1 (Contribution 7 and references [12,13]). Moreover, aptamers have proven to be very useful for the detection of small concentrations of VEGF for diagnostic purposes, in addition to therapeutic applications [14]. The application of aptamers for early detection and treatment of ovarian cancer is reviewed in Contribution 8. 

Small molecule inhibitors of the intracellular tyrosine kinase domains of the VEGFRs have been successfully developed for cancer treatment but have not yet reached the clinic for nAMD. These small molecules are generally less specific than macromolecules, and exhibit more side effects in systemic circulation [2,15]. For example, the triple VEGFR inhibitor tivozanib, which was recently approved to treat refractory advanced renal cell carcinoma, has side effects such as hypertension [16]. The underlying mechanism was studied in mice models in Contribution 9. On the other hand, the lower specificity of these small molecules allows multi-targeting, as demonstrated by chiauranib, an inhibitor of the VEGFR/Aurora B/CSF-1R kinases [17], and promising preclinical studies on transformed follicular lymphoma are presented in Contribution 10. 

The VEGF/VEGFR axis also plays a role in several pathologies other than cancers and retinopathies, such as diabetic nephropathy [18]. The underlying mechanism and potential therapeutic applications were investigated in rat models of diabetes treated with suramin in Contribution 11. 

We thank all authors and reviewers for their valuable contributions to this Special Issue, and we believe that this will trigger further studies to better understand the action of Pharmaceuticals targeting the key VEGF/VEGFR axis and develop a next generation of drugs, for improved patient outcomes. 
